# An integrated transcriptomics and metabolomics analysis of the *Cucurbita pepo* nectary implicates key modules of primary metabolism involved in nectar synthesis and secretion

**DOI:** 10.1002/pld3.120

**Published:** 2019-02-28

**Authors:** Erik M. Solhaug, Rahul Roy, Elizabeth C. Chatt, Peter M. Klinkenberg, Nur‐Aziatull Mohd‐Fadzil, Marshall Hampton, Basil J. Nikolau, Clay J. Carter

**Affiliations:** ^1^ Department of Plant & Microbial Biology University of Minnesota St. Paul Minnesota; ^2^ Roy J. Carver Department of Biochemistry, Biophysics & Molecular Biology Center for Metabolic Biology Iowa State University Ames Iowa; ^3^ Department of Mathematics & Statistics University of Minnesota Duluth Duluth Minnesota

**Keywords:** *Arabidopsis thaliana*, *Cucurbita pepo*, nectar, nectaries, nectary

## Abstract

Nectar is the main reward that flowers offer to pollinators to entice repeated visitation. *Cucurbita pepo* (squash) is an excellent model for studying nectar biology, as it has large nectaries that produce large volumes of nectar relative to most other species. Squash is also monoecious, having both female and male flowers on the same plant, which allows comparative analyses of nectary function in one individual. Here, we report the nectary transcriptomes from both female and male nectaries at four stages of floral maturation. Analysis of these transcriptomes and subsequent confirmatory experiments revealed a metabolic progression in nectaries leading from starch synthesis to starch degradation and to sucrose biosynthesis. These results are consistent with previously published models of nectar secretion and also suggest how a sucrose‐rich nectar can be synthesized and secreted in the absence of active transport across the plasma membrane. Nontargeted metabolomic analyses of nectars also confidently identified 40 metabolites in both female and male nectars, with some displaying preferential accumulation in nectar of either male or female flowers. Cumulatively, this study identified gene targets for reverse genetics approaches to study nectary function, as well as previously unreported nectar metabolites that may function in plant‐biotic interactions.

## 1 INTRODUCTION

Floral nectar is a sugary solution that serves as a reward for pollinators and is essential for successful reproduction in many flowering plants (Ollerton, Winfree, & Tarrant, 2011; Simpson & Neff, 1983). Diverse species produce nectar with different relative concentrations of sugars. Arabidopsis and other members of the Brassicaceae produce nectar that is rich in hexoses (glucose and fructose) (Davis, Fowke, Sawhney, & Low, 1996; Davis, Pylatuik, Paradis, & Low, 1998; Davis, Sawhney, Fowke, & Low, 1995), whereas tobacco and squash (*Cucurbita pepo*) produce nectar rich in sucrose (Nepi, Guarnieri, & Pacini, 2001). Squash flowers are hemitropous (Dmitruk & Weryszko‐Chmielewska, 2013) with the nectaries hidden but still accessible to pollinators. *C. pepo* is a monoecious species that produces both staminate and pistillate flowers, which both secrete nectar. Nectar secretion begins at around dawn and lasts nearly 6 hr (Nepi et al., 2001). Male and female flowers in *C. pepo* differ in the timing of nectar secretion, with male flowers increasing nectar secretion until ~3 hr post flower opening before leveling off, whereas in female flowers nectar levels continue to increase throughout the morning and until the flowers start to close (at ~6 hr post‐opening) (Nepi et al., 2001). It is likely that the difference in timing has biological significance as the reproductive success of a plant depends on the sequential visitation of pollinators to male flowers first to receive pollen before visiting female flowers for pollination. Also, on average female flowers produce more nectar, and it contains higher sugar content than the nectar produced by male flowers (Nepi et al., 2001). Nectar levels in both flower types decrease drastically by 6 hr post secretion, suggesting that some resorption of nectar occurs (Nepi et al., 2001).

Nectar secretion involves a number of steps that are intricately regulated in order to maximize pollination while not wasting resources (Heil, 2011; Pleasants & Chaplin, 1983). Floral nectaries in most species are non‐photosynthetic sink tissues that depend on photosynthate (e.g., sucrose) and other “pre‐nectar” components to be delivered via the vasculature, and these are often stored prior to secretion (Heil, 2011; Nepi & Stpiczynska, 2008; Pacini & Nepi, 2007). For example, high levels of starch accumulation in the parenchyma of immature nectaries (before secretion) has been reported for many flowering species (Lin et al., 2014; Peng, Li, Hao, Xu, & Bai, 2004; Ren, Healy, Horner, Martha, & Thornburg, 2007; Ren, Healy, Klyne, et al., 2007). This starch is absent from nectary tissues during and after secretion (Peng et al., 2004; Ren, Healy, Horner, et al., 2007; Ren, Healy, Klyne, et al., 2007; Zhu, Hu, & Müller, 1997), suggesting nectary starch may serve as a temporary carbon store to facilitate fast production of soluble sugars for nectar secretion. While starch accumulation and degradation are strongly correlated to nectar secretion in diverse plant species, the specific genes, proteins, and metabolites that are involved in this process have had limited study and only in *Nicotiana* spp. (Ren, Healy, Horner, et al., 2007; Ren, Healy, Klyne, et al., 2007).

After starch degradation, there is a well‐supported model for nectar synthesis and secretion in Arabidopsis (Lin et al., 2014; Roy, Schmitt, Thomas, & Carter, 2017). Specifically, starch breakdown products (chiefly maltose and hexose‐phosphates) are first assembled into sucrose by the action of sucrose‐phosphate synthases (SPS) and sucrose‐phosphate phosphatases, among other enzymes, whereupon the sucrose is exported from the nectary cells in a concentration‐dependent manner via the uniporter SWEET9. In some species that generate hexose‐rich nectars, the exported sucrose may be hydrolyzed into glucose and fructose by cell wall invertases (CWINV). Evidence for this model of nectar secretion is based on biochemical analyses (Liu & Thornburg, 2012; Ren, Healy, Horner, et al., 2007; Ren, Healy, Klyne, et al., 2007), in combination with the fact that genetic ablation of sucrose synthesis, export or extracellular hydrolysis all impair nectar secretion in Arabidopsis and/or tobacco (Lin et al., 2014; Ruhlmann, Kram, & Carter, 2010).

Additionally, several aspects of hormonal and transcriptional control of nectary functions have been studied in Arabidopsis and other species (Carter & Thornburg, 2003; Heil et al., 2001; Liu, Ren, Guirgis, & Thornburg, 2009; Liu & Thornburg, 2012; Radhika, Kost, Boland, & Heil, 2010; Wang, Liu, Niu, Timko, & Zhang, 2014). However, a majority of studies, particularly in Arabidopsis, have largely been dependent on genetic strategies, and biochemical confirmation of the conclusions have been hampered by the small size of Arabidopsis flowers and small amounts of nectar produced by these flowers. In contrast, plants in the *Cucurbita* genus, such as *C. pepo*, develop much larger flowers and produce >100‐fold more nectar per flower than Arabidopsis, and thus are more amenable to biochemical studies on nectar production [e.g., (Chatt et al., 2018; Nepi, Pacini, & Willemse, 1996; Nepi et al., 2001, 2012)].

Here, we expand the potential utility of *C. pepo* and related cucurbits as models to pursue physiological, genetic, and biochemical studies of nectar production through transcriptomic and metabolomics analyses of nectaries and nectar through the process of maturation. Subsequent experiments placed genes, pathways, and metabolites in a physiological context. This report represents an important step in improving our understanding of cucurbit nectary biology.

## 2 METHODS

### 2.1 Plant growth, tissue collection, RNA isolation and sequencing

*Cucurbita pepo* (Crookneck Yellow Squash) plants were grown on Sun Gro LC8 soil under a 16 hr day/8 hr night cycle, photosynthetic photon flux of 250 μmol m^−2^ s^−1^ at leaf level, and a temperature of ~23°C. Four types of RNA samples were separately prepared from manually collected nectaries of both male and female squash flowers, including: “pre‐secretory #1” (24 hr prior to anthesis/nectar secretion), “pre‐secretory #2” (15 hr prior to anthesis/nectar secretion), “secretory” (full anthesis, 2–3 hr after dawn), and “post‐secretory” (9 hr after the “secretory” stage). All nectary tissues were manually dissected by hand with the RNA being immediately extracted by mechanical disruption with a microcentrifuge pestle and using an RNAqueous^®^ RNA isolation kit (Ambion, Austin, TX, USA) with Plant RNA Isolation Aid (Ambion). Agarose gel electrophoresis and UV spectrophotometry were used to assess RNA quality for all samples prior to submission to the University of Minnesota Genomics Center for mRNA isolation, barcoded library creation and Illumina HiSeq 2500 sequencing. Twenty‐three TruSeq RNA v2 libraries were created (triplicate samples for male and female nectaries at four timepoints each, except for only duplicate samples of female “pre‐secretory #2” nectaries) and sequenced via 50 bp, paired‐end runs on the HiSeq 2500 using Rapid chemistry. All libraries were pooled and sequenced across two full lanes. This generated over 240 M reads for each lane and the average quality scores were above Q30.

### 2.2 Informatic analyses

The sequenced reads from nectary samples were assembled separately using Trinity (Grabherr et al., 2011), which automatically takes read quality and consistency into account, and yielded 70,111 contigs. This contig set was mapped to both the *Cucumis melo, Cucurbita pepo* and *Arabidopsis thaliana* genomes by NCBI's blastn (Altschul et al., 1990), with an E‐value cutoff of 0.00001, and the counts upper‐quartile normalized. *C. pepo* orthologs to *C. melo* genes were determined via blastn with an E‐value cutoff of 0.00001. Normalized counts were fitted to a negative binomial distribution using DESeq v1.6.1 (Anders & Huber, 2010). The resulting *p*‐values from DESeq were filtered by restricting to contigs with a 50% or greater change in mean expression between nectary stages. The Benjamini–Hochberg method was used to control the false discovery rate of contigs determined to be differentially expressed to 0.05 (Benjamini & Hochberg, 1995).

For gene ontology analyses, genes displaying >2‐fold and statistically significant differences (as determined by DESeq and FDR via the Benjamini–Hochberg method described above, *p* < 0.05) in various stages of only female, only male, or both nectaries combined were analyzed using the “Statistical Overrepresentation Test” tool via the PANTHER Classification System using the default settings and Bonferroni correction for multiple testing (*p* < 0.05) [(Mi et al., 2017) http://pantherdb.org/]. Information on biological processes, molecular function, and cellular component terms were extracted and saved in Microsoft Excel sheets. Heatmaps representing gene expression of candidate genes in male and female nectaries was performed using the Heml 1.0 (Heatmap Illustrator) software package. Fold‐change for each candidate gene for each stage was calculated with respect to the 0 hr stage. The ratios were then used by the Heml toolkit to generate heatmaps. Scales were kept the same for all genes analyzed across various stages of both male and female flowers.

### 2.4 Real time RT‐PCR (RNAseq validation)

The same RNA samples used for sequencing were also subjected to cDNA preparation using the Promega GoScript Reverse Transcription System (Catalog # A5000), with 5 μg of RNA used for cDNA preparation. Expression patterns for key genes that showed differential expression via RNAseq analyses were validated by quantitative RT‐PCR using Agilent Brilliant III Ultra‐fast SYBR Green QPCR Master Mix (Catalog #600882) and a cDNA template concentration of 1 ng/μl. Expression values are expressed as fold‐change relative to 0 hr timepoint and are based on the delta delta Ct values obtained from the normalized Ct values for each gene. Gene expression was normalized to a gene encoding a RING/U‐Box ligase superfamily protein (*C. pepo* hit = gi|LOC111790689|ref|XM_023671703.1|). This gene was chosen as the internal reference based on its stable expression level in all nectary samples in our RNA‐seq dataset. Primer sequences for each gene are provided in Table S1.

### 2.5 Lugol staining

Male and female squash flowers were bisected longitudinally with a scalpel. The flower halves were dipped in 0.05 molar iodine/potassium iodide stain (Fischer Scientific Cat#S93408) for 60 s. They were subsequently washed twice for 2 min each in 50 ml of water to remove excess stain and imaged using a dissecting microscope. Care was taken not to have an air bubble trapped in the nectary cup as the flower was dipped in the lugol solution.

### 2.6 Quantitative starch assay

Total starch was quantitatively determined using the Megazyme Total Starch Assay Kit Analysis (Megazyme, Cat# K‐TSTA). Samples were ground in 0.25 ml of 80% ethanol, then an additional 0.25 ml of 80% ethanol was added. Samples were incubated at 80°C for 5 min in order to remove soluble sugars and maltodextrins. Samples were centrifuged for 10 min at 1,800 *g* and supernatants were removed. The samples were resuspended in 1 ml 80% ethanol followed by repeating centrifugation at 4,300 rpm for 10 min and discarding supernatant. About 300 μl of α‐amylase (100 U/ml in 100 mM sodium acetate buffer, pH 5.0) was added to each sample, followed by incubation at 100°C for 6 min with vortexing at 2 min intervals. Ten microliters of amyloglucosidase (3,300 U/ml) was then added to each sample and samples were incubated at 50°C for 30 min. Samples were then made up to 1 ml final volume then centrifuged at 900 *g*. Supernatants were then assayed for D‐glucose using the glucose oxidase/peroxidase (GOPOD) method. The −24 hr and −15 hr samples were diluted 10‐fold in order to get the concentration of D‐glucose in the range of detection for the GOPOD assay. 33 μl of supernatant was added to 1 ml of GOPOD solution (made according to kit protocol) in duplicate and tubes were incubated at 50°C for 20 min. Absorbance was read at 510 nm using a visible spectrophotometer. Starch content (% w/w) was calculated using the equation for solid samples provided in the megazyme protocol supplied with the kit.

### 2.7 Amylase activity assays

Nectary tissue was harvested at all four stages of nectar secretion and stored at −80°C before use. Samples were ground in 150 μl Tris‐HCl, pH 8.0 on ice then centrifuged at max speed for 20 min at 4°C. Ten microliter of supernatant was added to a new tube containing 150 μl of 0.1 M sodium acetate (pH 4.6) and 75 μl of amylopectin (20 mg/ml in 0.2 M KOH). Each reaction was then made up to 300 μl with deionized water and then incubated at 37°C for 60 min. The reactions were ceased by incubation at 100°C for 3 min. Twenty microliter of each reaction was added to 750 μl of p‐hydroxybenzoic acid hydrazide (PAHBAH) solution and DI water was added to a final volume of 1 ml. Samples were then incubated at 100°C for 5 min then absorbance at 410 nm was read for each sample blanked against a reagent blank with Tris‐HCl grinding buffer added in the place of crude protein. Amylopectin stocks were prepared by boiling amylopectin/KOH solution for 10 min until the solution became clear. Then aliquots were stored at −20°C prior to use. PAHBAH solution was prepared by mixing 1 part 5% p‐hydroxybenzoic acid hydrazide in 0.5 M HCl with four parts 0.5 M NaOH.

### 2.8 Soluble sugar assay

Soluble sugars were quantified using an Amp‐red/glucose oxidase/horseradish peroxidase method coupled with endogenous invertase activity as previously described (Bender et al., 2012; Ruhlmann et al., 2010). In brief, nectary tissue from each stage was ground in protein extraction buffer then subjected to 20 min treatment with and without the addition of 10 mM sucrose. Reactions were diluted 1:100 to get into the working range of the glucose assay. The glucose assay mix (Ruhlmann et al., 2010) consisted of 38.5 μM Amp‐red (stock solution dissolved in DMSO), 5 units of horse radish peroxidase and glucose oxidase, and 33 mM sodium phosphate buffer pH 7. About 25 μl of assay solution was mixed with 75 μl of diluted sugar solution and absorbance was measured at 570 nm for each sample. Values were compared to a standard curve with glucose to determine the absolute amount of soluble sugar in each sample.

### 2.9 Nectar metabolomics

Two separate GC‐MS‐based methods were employed for untargeted metabolite profiling of six nectar samples from independent male and female flowers of *C. pepo*. The first of these provided data on the predominant sugars that constitute the nectar (i.e., sucrose, glucose, and fructose). Specifically, 1 μl of nectar was spiked with 10 μg ribitol as an internal standard, and the mixture was dried overnight by lyophilization. The sample underwent methoximation at 30°C for 90 min while continuously shaking with 20 mg/ml methoxyamine hydrochloride dissolved in pyridine. The methoximated sample was silylated for 30 min at 60°C with N,O‐Bis(trimethylsilyl)trifluoroacetamide (BSTFA)/1% trimethylchlorosilane (TMCS). Following dilution with 1.5 ml pyridine, 1 μl of sample was analyzed by GC‐MS.

Less abundant constituents of the nectar were extracted from a 5‐μl aliquot of nectar sample that was spiked with 0.5 μg nonadecanoic acid and 1 μg ribitol as internal standards. Hot methanol (2.5 ml) was immediately added to the nectar, and the mixture was incubated at 60°C for 10 min. Following sonication for 10 min at 4°C, chloroform (2.5 ml) and water (1.5 ml) were sequentially added, and the mixture was vortexed. Centrifugation separated the polar and nonpolar fractions, and the entire nonpolar fraction and half of the polar fraction was recovered to separate 2 ml screw‐cap glass vials and dried overnight by lyophilization. The polar fraction underwent methoximation as previously described, and both polar and nonpolar fraction were silylated for 30 min at 60°C with BSTFA/1% TCMS.

The derivatized metabolites (the sugars, polar, and nonpolar fractions) were analyzed using an Agilent Technologies Model 7890A GC system equipped with an HP‐5 ms (30 m, 0.25 mm, 0.25 μm) GC column that was coupled to an Agilent Technologies 7683B series injector and Agilent Technologies Model 5975C inert XL MSD with Triple‐Axis Detector mass spectrometer. Chromatography parameters for the polar and nonpolar fractions were set to a helium gas flow rate of 1 ml/min, 2 μl injection, with a temperature gradient of 80°C–320°C increasing at a rate of 5°C/min, followed by a 9‐min hold at 320°C. The polar fractions were analyzed using a “heart‐cut” method (Boeker, Leppert, Mysliwietz, & Lammers, 2013) which diverted gas flow to an FID detector during elution times for fructose, glucose, and sucrose.

GC parameters for predominant sugar metabolites were set to a helium gas flow rate of 1 ml/min, 1 μl injection with a 10:1 split, and a temperature gradient of 100°C–180°C increasing at a rate of 15°C/min, then 5°C/min to 305°C, 15°C/min to 320°C, followed by a 5‐min hold at 320°C. Deconvolution and integration of resulting spectra was performed with AMDIS (Automated Mass Spectral Deconvolution and Identification System) software. Analyte peaks were identified by comparing mass spectra and retention indices to the NIST14 Mass Spectral Library and authentic standards when possible to confirm identification.

## 3 RESULTS

### 3.1 Experimental design

**Figure 1:**
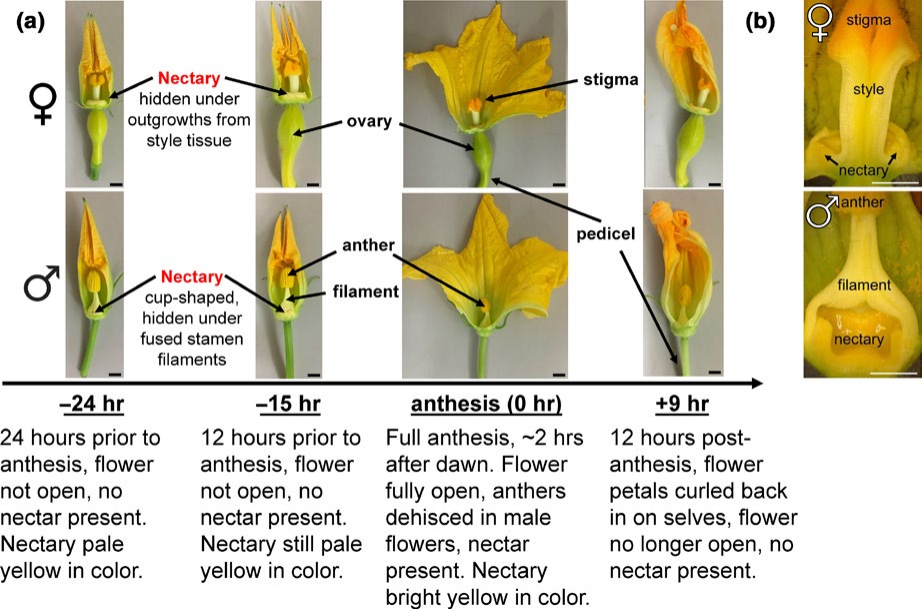
Developmental stages and tissues used for squash nectar/y analyses. (a) Timepoints used for female (top) and male (bottom) nectary collections. (b) Cross‐section of female and male flowers to reveal nectaries. Scale bar equals 5 mm

To understand the nexus of genes and processes that are activated in *C. pepo* nectaries throughout maturation, we decided to take a transcriptomic strategy. This approach was previously used with Arabidopsis nectaries (Kram, Xu, & Carter, 2009) and led to the identification of key genes that regulate nectary function and nectar production [e.g., (Bender et al., 2012, 2013; Kram & Carter, 2009; Lin et al., 2014; Ruhlmann et al., 2010; Schmitt, Roy, Klinkenberg, Jia, & Carter, 2018; Wiesen et al., 2016)]. Squash nectaries are considerably larger than Arabidopsis (~1 cm diameter for squash vs. ~100 μm for Arabidopsis) and produce relatively large amounts of nectar (>50 μl for squash vs. ≪1 μl for Arabidopsis, per flower). A transcriptomic understanding of the squash model would thus further the field of nectar biology, as physiological, biochemical, and cellular studies in downstream experiments would be made easier than with other species. We proceeded with an experimental plan where four stages of both pistillate (female) and staminate (male) flowers were selected to study nectary gene expression as affected by maturation (Figure 1).

The developmental stages chosen for analyses centered around anthesis, which is when pollen is released by anthers. Nectar production is synchronized with anthesis (Nepi et al., 1996, 2001) and hence this study was centered around the time of anthesis, which aided the uniform timing of tissue collection for subsequent studies. Squash flowers progress from small, closed green buds to open flowers relatively rapidly (within a few days), and we tracked their progress through key visual cues. The “pre‐secretory stage 1” occurs 24 hr before anthesis, and was recognized by the fact that buds are still closed but have a yellow coloration at the tips of the fused corolla; this stage is designated as the “−24 hr” timepoint (Figure 1a). We noted that 9 hr later the yellow color intensifies and the tip of the corolla is slightly unfurled and we designated this as “pre‐secretory stage 2” or the “−15 hr” timepoint (~12 hr before dawn). Open flowers actively producing nectar (~3 hr after dawn) were termed “secretory stage” or the “0 hr” timepoint, and 9 hr later (~12 hr after dawn), when the flowers had closed, was chosen as “post‐secretory” or “+9 hr” timepoint. While nectar was completely absent from the flowers at the −24 and −15 hr timepoints, copious amounts of nectar were present in the 0 hr and +9 hr flowers. Replicate RNAs were isolated from the nectaries (e.g., Figure 1b) at each of these timepoints and subjected to Illumina‐based RNA‐seq analyses.

### 3.2 RNA sequencing and differential expression analyses

Over 240M reads (50 bp, paired end) derived from squash nectary RNAs were assembled into 70,111 contigs and initially mapped to both the *C. melo* (melon) and *Arabidopsis thaliana* Col‐0 genomes, with the corresponding *C. pepo* orthologs [from the recently published zucchini genome (Montero‐Pau et al., 2018)] to the *C. melo* genes subsequently being incorporated into the analysis. The rationale for mapping contigs to melon is that it is a close relative to squash with a fully sequenced genome; similarly, contigs were mapped to Arabidopsis because it has a very well annotated genome and has served as the genetic model for plant biology, including the process of nectar production [reviewed in (Roy et al., 2017)]. Altogether, *C. pepo* nectary contigs mapped to 8,863 unique *C. melo* and *C. pepo* genes (Table S1), which were subsequently used for all downstream analyses of differential expression.

**Figure 2:**
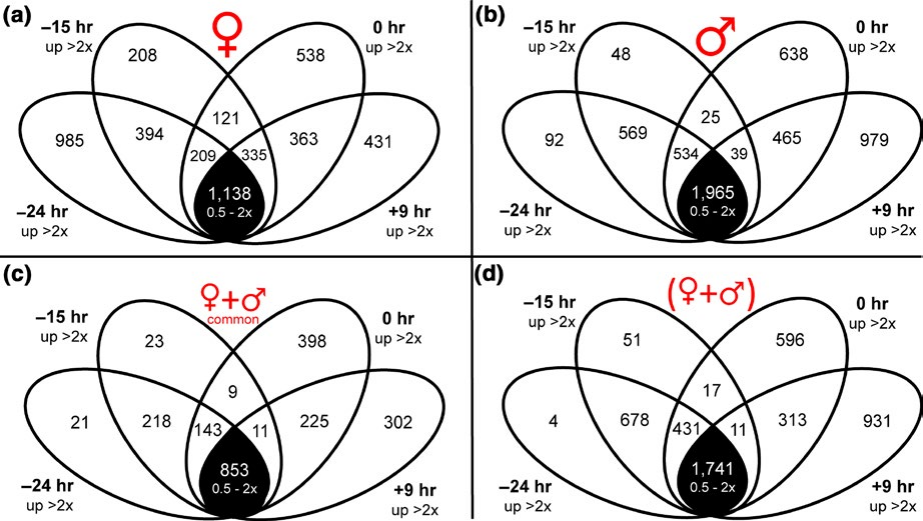
Venn diagram set‐representation of differentially expressed genes in nectaries associated with developmental stages. Differentially expressed genes are defined as those showing a > 2‐fold‐change in expression in any pairwise or multistage comparison (*p* < 0.05). The number of genes that fail this test (commonly expressed at all timepoints) are identified in the black‐shaded subset. (a) Number of genes upregulated in female nectaries at the −24 hr, −15 hr, 0 hr or +9 hr timepoints in female nectaries. (b) Number of genes upregulated in male nectaries at the −24 hr, −15 hr, 0 hr or +9 hr timepoints. (c) Number of genes commonly upregulated in both male and female nectaries (i.e., differentially expressed genes appearing at the same timepoints in both a and b) at the −24 hr, −15 hr, 0 hr or +9 hr timepoints. (d) Number of genes upregulated at each developmental stage when reads from both female and male nectaries from a particular stage are analyzed together as a single unit

Differentially expressed genes between nectary stages were defined as having a statistically significant *and* mean twofold difference in expression. The lists of differentially expressed genes between stages in female and male nectaries are available in Appendix S2, respectively. We additionally performed a separate differential expression analysis where reads from both female and male nectaries were grouped together by developmental stage (Appendix S2). These analyses revealed genes that are upregulated at specific stages of maturation, as well as ones that were commonly expressed at all timepoints in both male and female flowers (Figure 2, Appendix [Link], [Link], [Link], [Link]). The numbers of genes displaying stage‐dependent upregulation in nectary expression are indicated in the Venn diagrams for female (Figure 2a; *n* = 3 for each stage, except *n* = 2 for −15 hr), male (Figure 2b, *n* = 3 for each stage), female and male together when analyzed for significance separately (Figure 2c; i.e., differentially expressed genes commonly appearing in both Figure 2a,b), and female and male together when all reads for a given stage were analyzed as a group for fold‐change and statistically significant differences in expression (Figure 2d; *n* = 6 for −24, 0 and +9 hr, *n* = 5 for −15 hr). The nonoverlapping regions of each Venn diagram indicate the number of genes that are upregulated at a specific developmental stage (i.e., at the −24 hr, −15 hr, 0 hr or +9 hr timepoints), whereas the numbers shown in overlapping regions are commonly upregulated at two or more timepoints over the others. For example, in female nectaries (Figure 2a) there were 394 genes expressed >2‐fold higher (and statistically significant) at both the −24 and −15 hr timepoints over the 0 and +9 hr timepoints; similarly, there were 209 genes that were more highly expressed at the −24, −15, and 0 hr timepoints over the +9 hr stage.

### 3.3 Validation of RNA‐seq data

**Figure 3:**
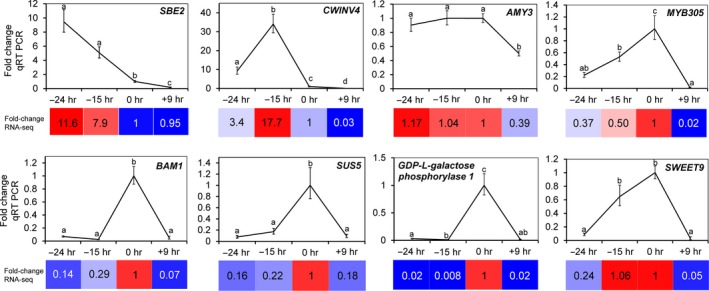
Validation of RNA‐seq data. qRT PCR was used to validate the expression patterns of select differentially expressed genes in male nectaries identified through RNA‐seq analyses. The fold‐change in each stage was determined relative to 0 hr (the secretory stage [anthesis] was set as 1). Statistically significant differences in expression between the stages are noted by different letters (pairwise, two‐tailed *t* test, *p* < 0.05). The colored boxes represent the corresponding fold‐change observed by RNA‐seq analysis. SBE2 = Starch Branching Enzyme 2, CWINV4 = Cell Wall Invertase 4, AMY3 = Alpha‐amylase 3, BAM1 = Beta‐amylase 1, SUS5 = Sucrose Synthase 5. MYB305 and SWEET9 are the full gene names

The differential expression identified through the RNA‐seq analyses was subsequently validated by qRT‐PCR using RNA isolated from male nectaries (Figure 3). The genes chosen for qRT‐PCR validation primarily included those predicted to be involved in carbohydrate metabolism and/or orthologs of genes known to be involved in nectary function. A majority of the selected genes displayed upregulation at one or more specific timepoint. For example, *Starch Branching Enzyme 2* (*SBE2*), encoding a protein predicted to be involved in starch synthesis, displayed high expression at the −24 hr timepoint, and declined at each subsequent developmental timepoint. Conversely, a gene associated with starch breakdown, β*‐amylase 1* (*BAM1*), displayed low expression level at every developmental stage except for the 0 hr timepoint. Presumptive orthologs of several genes known to be required for nectar production in other species—*MYB305*,* SWEET9*, and *CWINV4* (Lin et al., 2014; Liu & Thornburg, 2012; Liu et al., 2009; Ruhlmann et al., 2010; Schmitt et al., 2018; Wang et al., 2014)—also displayed stage‐specific induction. Both the *MYB305* and *SWEET9* transcripts displayed low expression at the −24 hr timepoint and peaked at the 0 hr timepoint, whereas *CWINV4* displayed highest expression at the −15 hr timepoint.

### 3.4 Gene ontology analyses identify a shift from anabolic to catabolic processes

Genes displaying stage‐enriched expression (from Figure 2A,B,D [female, male and female/male together, respectively]) were subjected to gene ontology (GO) analyses to identify processes and metabolic pathways overrepresented in nectaries during a specific stage of development (Appendix [Link], [Link], [Link]). At the −24 hr timepoint, female nectaries displayed significant enrichment for anabolic processes (see *Biological Processes* and *Molecular Function* tabs in Appendix S7), notably transcripts encoding proteins involved in amino acid biosynthesis, transcription, and translation processes are overrepresented. However, by −15 hr, a distinct shift toward catabolic processes was noted in female nectaries, particularly with regard to starch degradation. The secretory nectaries (0 hr timepoint) displayed an extension of catabolic processes to amino acids. Unique GO terms enriched at the post‐secretory female nectaries (+9 hr timepoint) included the appearance of those related to senescence, including assembly of the autophagosome.

Interestingly, male nectaries displayed no significant enrichment of genes related to specific GO terms at either the −24 or −15 hr timepoints, which could be due to the relatively few number of genes being upregulated specifically at either stage as compared to female nectaries (Figure 2). As such, we analyzed genes commonly upregulated at the −24 and −15 hr timepoints over 0 and +9 hr (i.e., the 569 genes represented in the overlapping region of −24 and −15 hr in Figure 2b, Appendix S8). This analysis identified both starch biosynthetic and catabolic processes as being highly enriched in pre‐nectar secretion stages (i.e., −24 and −15 hr timepoints). Enriched GO terms at the secretory (0 hr) and post‐secretory (+9 hr) timepoints of male flowers were similar to those identified in female nectaries. The GO analysis of combined reads for both male and female nectaries (from Figure 2d) closely mirrored such analyses of the male nectaries (Appendix S9).

### 3.5 Carbohydrate and nectar secretion‐related processes

**Figure 4:**
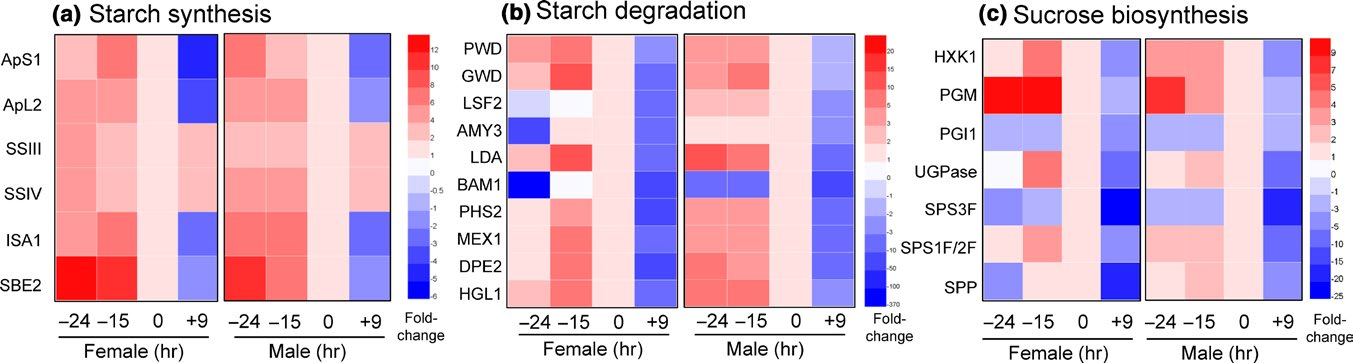
Expression analysis of genes involved in starch and sugar metabolism. Normalized RNA‐seq data was used to generate heat maps for nectary‐expressed genes involved in starch synthesis (a), starch degradation (b), and sucrose biosynthesis (c). The fold‐change in each stage was determined relative to 0 hr (the secretory stage [anthesis] was set as 1). In general, starch biosynthesis genes peak at −24 hr, starch degradation genes peak at −15 hr (with the exception of BAM1), and sucrose biosynthesis genes are highest at −15 and 0 hr. This analysis supports a progression of starch synthesis, starch degradation, and sucrose biosynthesis as being essential for nectary function. Full names for the abbreviations of individual genes are provided in Appendix S10

The role of starch and sugar metabolism is well characterized in nectar production (Nepi et al., 1996, 2001; Ren, Healy, Horner, et al., 2007; Ren, Healy, Klyne, et al., 2007), and this is clearly reflected in the GO analyses of the nectary transcriptomics data. The RNAseq data were characterized to identify regulation of carbohydrate interconversions as indicated by stage‐specific expression of candidate genes involved in canonical biosynthetic or catabolic processes (Figure 4, Appendix S10). We grouped genes based on their involvement in starch synthesis starting from ADP‐glucose and ending in starch (Figure 4a), starch degradation ending in glucose (Figure 4b, genes were chosen based on those outlined in Streb & Zeeman, 2012), and sucrose synthesis starting from glucose and including all genes required to convert glucose into sucrose (Figure 4c). The expression of genes involved in starch synthesis primarily peaked at the −24 hr timepoint (e.g. *CpSBE2* in Figures 3 and 4). There was a stark transition toward starch degradation between the −15 and 0 hr timepoints, with most degradative genes having highest expression at −15 hr, the notable exception being *CpBAM1*, which was strongly induced at the 0 hr timepoint (Figures 3 and 4b).

Sucrose synthesis coincides with starch degradation in the current model of nectar secretion (Lin et al., 2014; Roy et al., 2017). Indeed, transcript for the sucrose biosynthetic gene *Sucrose‐phosphate synthase 2* (*CpSPS2*) in female nectaries was relatively low at −24 hr, with high levels being observed at both −15 and 0 hr; however, in male nectaries *CpSPS2* expression was relatively constant at the −24, −15 and 0 hr timepoints in male nectaries, with a sharp drop‐off post‐secretion (+9 hr) (Figure 4c). Expression of other sucrose biosynthetic genes generally peaked at the −15 and 0 hr timepoints (Figure 4c), with one notable exception being *Phosphoglucomutase* (*CpPGM*), which was high at −24 hr, similar to other starch synthesis genes. PGM is involved in producing glucose‐1P for both starch (via ADP‐glucose) and sucrose synthesis (via UDP‐glucose), and is likely important for both of these processes in *C. pepo* nectaries (Streb & Zeeman, 2012).

Not represented in the carbohydrate metabolic pathways presented in Figure 4 are genes involved in establishing and maintaining sink status. Raffinose‐family oligosaccharides (RFOs) are the primary transport sugars in cucurbits (Zhang, Tolstikov, Turnbull, Hicks, & Fiehn, 2010), which are hydrolyzed in sink tissues by α‐galactosidases, releasing galactose and sucrose for further metabolism (Carmi et al., 2003). Indeed, two genes encoding putative α‐galactosidases, *Raffinose Synthase 6* (*CpRAFS6*), and *Seed Imbibition 2* (*CpSIP2*) are highly expressed in nectaries at −24 and −15 hr (Appendix [Link], [Link]), suggesting they are important for maintaining sink status during the starch filling stages.

The last two steps of nectar secretion in the current model are sucrose export via SWEET9 and extracellular hydrolysis by cell wall invertases. For the sucrose export step, *CpSWEET9* transcript began low at −24 hr and peaked at the 0 hr timepoint in both male and female nectaries (Figure 3). However, the most highly expressed cell wall invertase in squash nectaries, *CpCWINV4* (*Cell Wall Invertase 4*), the apparent ortholog to Arabidopsis *AtCWINV4* required for nectar secretion (Ruhlmann et al., 2010), displayed a unique timing of expression relative to other species. Specifically, its expression peaked at −15 hr (instead of at anthesis like other species), with almost no detectable transcript at either the −24 or 0 hr timepoints. Lastly, with regard to regulation of nectar secretion, *CpMYB305* is the ortholog of a transcription factor intimately tied to regulating carbohydrate metabolism in *Nicotiana* and Arabidopsis nectaries (Liu & Thornburg, 2012; Liu et al., 2009; Schmitt et al., 2018; Wang et al., 2014); it displayed highest expression at the 0 hr timepoint in both female and male nectaries (Figure 3).

**Figure 5:**
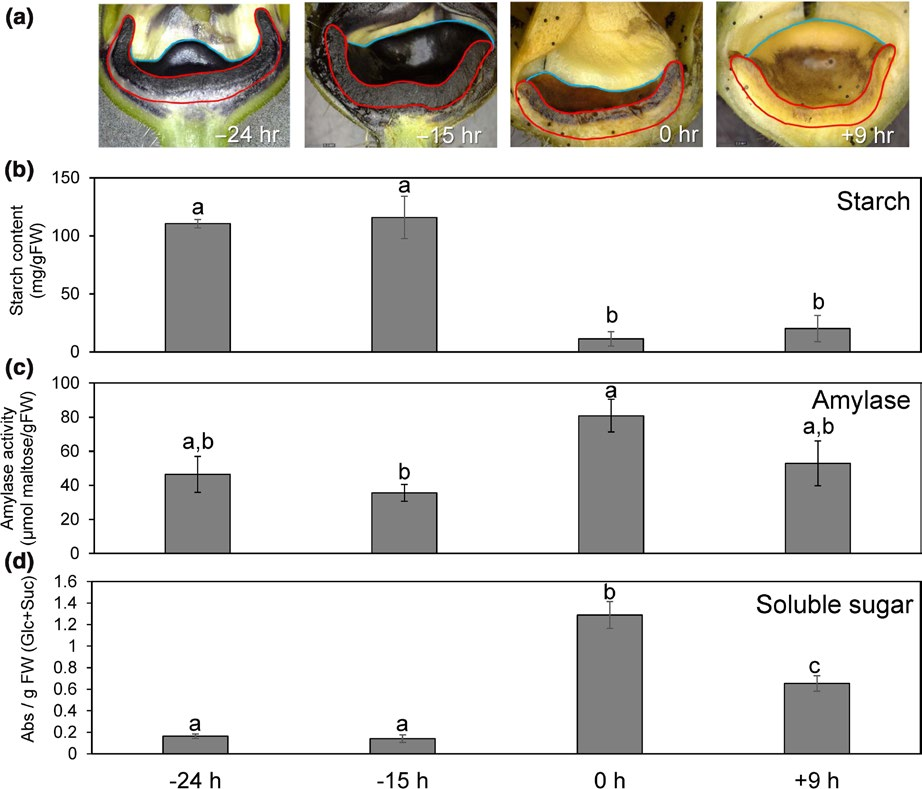
Starch and sugar metabolism in male squash nectaries at different developmental stages. (a) Longitudinally sectioned male squash flowers stained for starch accumulation with Lugol's iodine solution at different developmental stages. The face of the cut nectary surface is outlined in red, whereas the remaining intact bowl‐shaped nectary is outlined in blue. Scale bar equals 5 mm. (b) Quantitative determination of starch in squash nectaries. (c) Amylase activity present in squash nectaries at each stage. (d) Total soluble sucrose and glucose present in nectary tissues at each stage. Statistically significant differences between the stages are noted by different letters (pairwise, two‐tailed *t* test, *p* < 0.05)

The biological relevance of carbohydrate metabolism‐related gene expression patterns was partially validated by examining starch and soluble sugar accumulation patterns and conducting enzymatic assays (Figure 5). The −24 hr and −15 hr nectary stages displayed intense iodine staining (indicative of starch) and averaged ~10%–15% wt/wt starch on a fresh weight basis (Figure 5a,b). This starch was largely absent in secretory nectaries (0 hr) and remained low at +9 hr (Figure 5a,b). Consistent with a degradative mechanism, the observed decrease in starch content coincided both with an induction of total amylase activity (Figure 5c) and the higher accumulation of soluble sugars (sucrose and glucose) in the nectaries at the 0 hr timepoint (Figure 5d).

### 3.6 Transcriptional differences between female and male nectaries

Male and female flowers display a slightly different timing of nectar secretion and amount of nectar made (female flowers generally open a little later than male flowers and also produce more nectar (Nepi et al., 2001). As such, DESeq analysis was used to identify differentially expressed genes between female and male flowers specifically at the 0 hr (secretory) timepoint (Appendix S11). This analysis identified 134 genes more highly expressed in female over male nectaries at the 0 hr timepoint. In particular, the top three genes more highly expressed in female nectaries were all transcription factors, including *SHI‐related sequence 1*,* heat stress transcription factor A‐6b,* and *UNUSUAL FLORAL ORGANS‐like*. Conversely, male nectaries at the 0 hr timepoint expressed 43 genes more highly than their female counterpart.

### 3.7 Nectar metabolite analyses

Nectars not only contains sugars, but also have many solutes with biologically important roles (Adler & Irwin, 2012; Elliott, Irwin, Adler, & Williams, 2008; Irwin & Adler, 2008; Irwin, Adler, & Agrawal, 2004; Nicolson & Thornburg, 2007; Richardson et al., 2015). Nectar from both female and male flowers were characterized via a nontargeted metabolomic strategy (results shown in Table S2). Not surprisingly, sucrose (~2.1M), glucose (~0.3M), and fructose (~0.3M) were the primary metabolites observed in both male and female nectars, with a sucrose‐to‐hexose ratio of ~4:1 in female nectar and ~3.4:1 in male nectar. Lesser levels of other sugars and sugar alcohols were also observable, including inositol, maltose, galactose, arabinose, erythritol, erythrofuranose, ribose, xylose, xylitol, seduheptalose, and mannose (Table S2). Additional metabolites identified in both male and female nectars included amino acids and other polar compounds, such as 2,3‐butanediol, ethylene glycol, and the neurotransmitters gamma‐hydroxybutyric acid (GHB) and gamma‐amino butyric acid. Not surprising for an aqueous biological medium, nonpolar metabolites were fewer and of lower concentrations, these included 4‐coumaryl alcohol; 4‐hydroxybenzyl alcohol (gastrodigenin); 2‐thiophenecarboxylic acid, 4‐methoxyphenyl ester; and 4‐(hydroxymethyl)‐2‐methoxyphenol.

**Figure 6:**
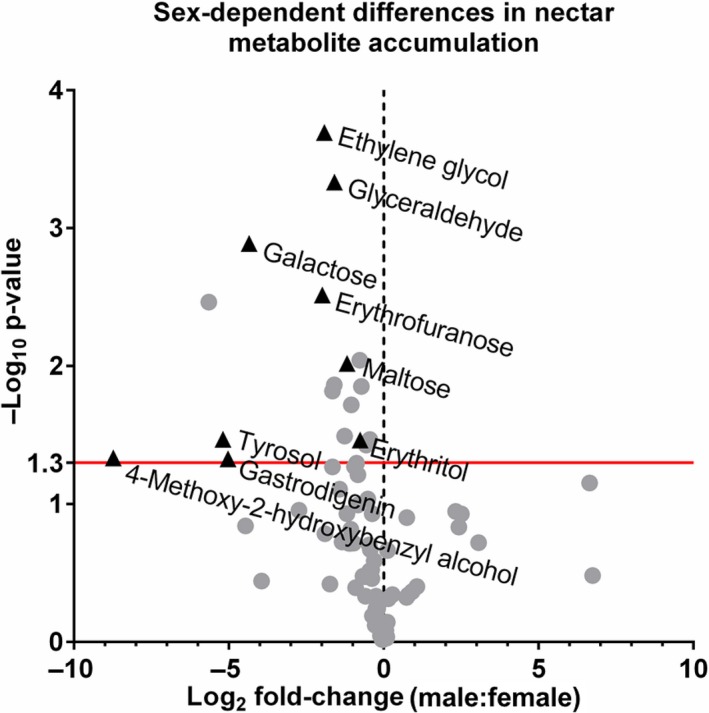
Volcano plot of the *Cucurbita pepo* nectar metabolome with the x axis representing the log_2_ fold‐change of male‐to‐female metabolite concentration and the *y*‐axis representing the negative log_10_ of the adjusted *p*‐value. Points above the red line represent metabolites with *p*‐values < 0.05 between male and female metabolite

A handful of these metabolites differentially accumulate between male and female nectars (Figure 6, Table S2), with most preferentially accumulating in the male nectar (~2–4‐fold higher levels), including: galactose, ethylene glycol, glyceraldehyde, erythrofuranose, maltose, erythritol, and tyrosol. While not statistically significant, one non‐proteinaceous amino acid, gamma‐amino butyric acid (GABA), occurs at fivefold higher level in female nectar over male nectar (Figure S1). The transcript of a putative GABA exporter (ortholog of *C. melo* XM_008448565.1) displayed a parallel increased level of accumulation in female nectaries at the secretory stage (0 hr timepoint) (Figure S1), which could account for the observed differences in GABA levels between male and female nectar.

## 4 DISCUSSION

This study represents the first report of global gene expression profile of cucurbit nectaries. While Arabidopsis and *Nicotiana* spp. have been extensively studied as genetic models for nectar production (Bender et al., 2012, 2013; Carter, Graham, & Thornburg, 1999; Carter, Shafir, Yehonatan, Palmer, & Thornburg, 2006; Carter & Thornburg, 2000, 2003, 2004a,2004b,2004c; Carter et al., 2007; Hampton et al., 2010; Horner et al., 2007; Kram & Carter, 2009; Kram et al., 2009; Lin et al., 2014; Liu & Thornburg, 2012; Liu et al., 2009; Naqvi et al., 2005; Ren, Healy, Horner, et al., 2007; Ren, Healy, Klyne, et al., 2007; Roy et al., 2017; Ruhlmann et al., 2010; Stitz, Hartl, Baldwin, & Gaquerel, 2014; Thomas, Hampton, Dorn, Marks, & Carter, 2017; Thornburg et al., 2003; Wiesen et al., 2016), an expansion of molecular biology approaches into other systems with larger nectaries (Figure 1) that produce copious amounts of nectar will aid our understanding of nectary biology, particularly with regard to quantitative biochemical, physiological, and comparative studies. Our study has revealed a plethora of squash genes and metabolic processes that are temporally regulated as the nectary progresses from pre‐secretion to secretion to post‐secretion stages of development.

Squash nectary RNAseq reads mapped to 8,863 unique *C. pepo* and *C. melo* genes, which is consistent with the number of genes identified as being expressed in Arabidopsis nectaries (9,066) and pennycress (12,335) nectaries (Kram et al., 2009; Thomas et al., 2017). Many genes displayed preferential expression at one or more stages of nectary maturation (Figure 2), with the data being validated by qRT‐PCR analysis of eight targeted genes (Figure 3); demonstrating the quality and utility of the RNAseq dataset for downstream analyses.

**Figure 7:**
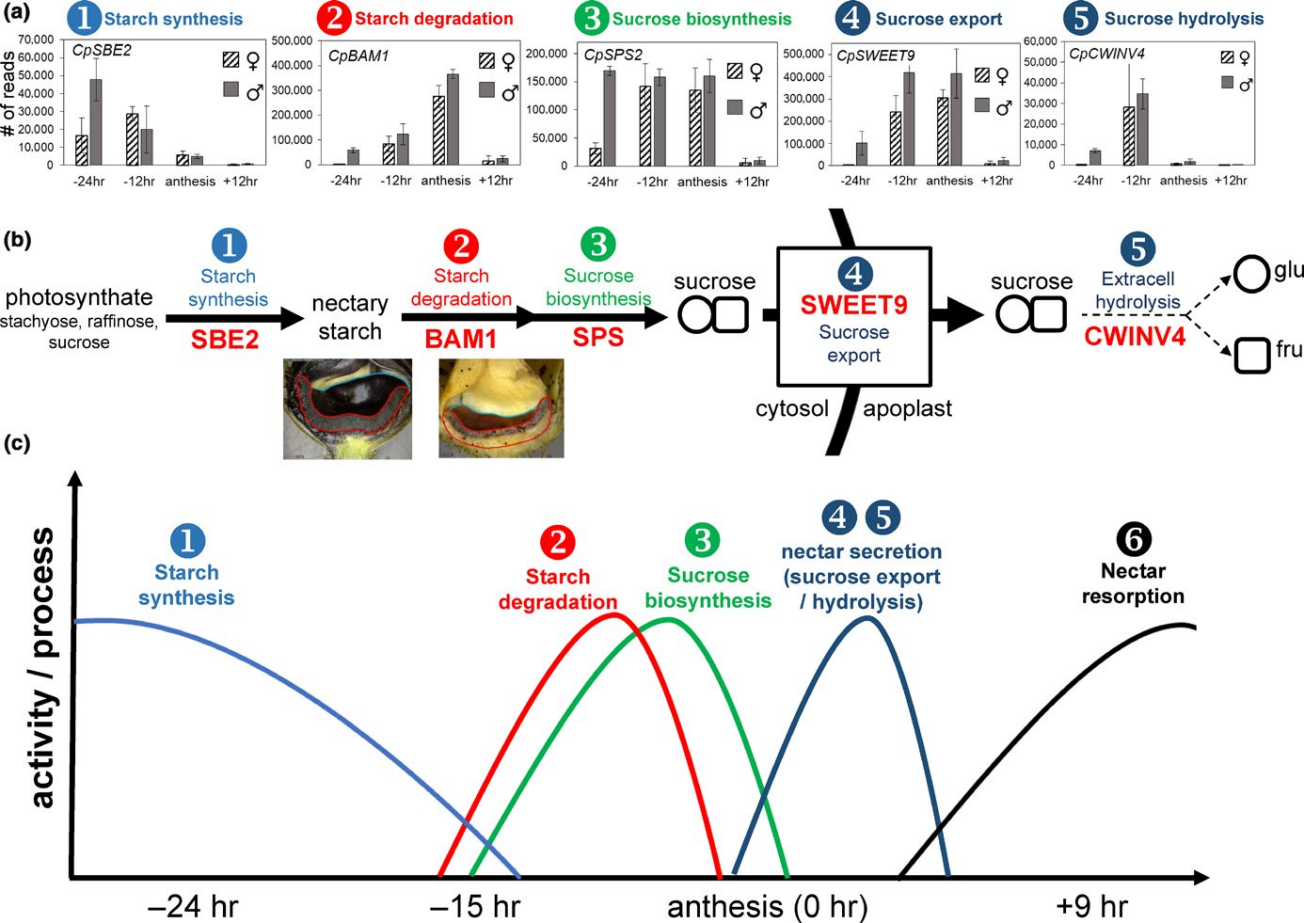
Representative genes and processes putatively involved in carbon flux in squash nectaries. (a) Expression of genes in male and female nectaries involved in each of the proposed steps of nectar synthesis and secretion as outlined in panel b; SBE = starch branching enzyme; BAM1 = beta‐amylase; SPS = sucrose‐phosphate synthase; SWEET9 = sucrose transporter; CWINV4 = cell wall invertase (c) Approximate timing of processes involved in nectar production based on data from this study and previously published reports

Analysis of our transcriptome data readily identified representative candidate genes displaying the appropriate timing of expression and/or activities associated with each of the proposed steps of nectar synthesis described in previous sections, including starch synthesis, starch degradation, sucrose synthesis, and sucrose export (Figures 3, 4, 5, with a summarized model in Figure 7). For example, expression of the starch biosynthesis gene *Starch Branching Enzyme2* (*CpSBE2*) was highest at −24 hr and steadily decreased thereafter, which was inversely proportional to *BAM1* expression (Figures 3, 4 and 7b). In terms of maintaining sink status, there are many proteins involved in ensuring a continuous supply of photosynthate to sink tissues. There have been a few studies on carbon partitioning and resource allocation in developing cucurbit fruits (Schapendonk & Brouwer, 1984), however, little is known about the regulation of sink–source relations in *C. pepo* nectaries. Cucurbits, such as squash and cucumber, transport photosynthate in the form of raffinose‐family oligosaccharides (RFOs) loaded and unloaded into the phloem predominantly via a symplastic route. In our study, we identified two very highly expressed α‐galactosidases (*CpRAFS6* and *CpSIP2*) that are likely involved in establishing and maintaining sink status in nectaries.

While the expression profiles for each of the genes and steps for nectar secretion in this study are largely consistent with what has been observed in other species [reviewed in (Roy et al., 2017)], a notable exception was *CpCWINV4*. In both Arabidopsis and pennycress nectaries, *SWEET9* and *CWINV4* have nearly identical read counts (Bender et al., 2013; Kram et al., 2009; Lin et al., 2014; Ruhlmann et al., 2010; Thomas et al., 2017); however, in squash, *CpCWINV4* is expressed in low quantities (maximum of ~30,000 reads) in nectaries relative to *CpSWEET9* (~400,000 reads) (Figure 7a). In contrast to Arabidopsis, *C. pepo* flowers produce nectar rich in sucrose (Nepi et al., 2001), which necessitates a reduction in extracellular sucrose hydrolysis after sucrose export from the cell. Thus, *CpCWINV4* probably does not play the same role in squash as *AtCWINV4* does in Arabidopsis (i.e., in generating a concentration gradient to drive sugar export); however, since squash nectar contains ~20% hexoses, its activity likely is important in dictating final nectar quality. It should also be noted that in sunflower a reduction in nectary CWINV expression led to an increase in nectar sucrose (Prasifka et al., 2018).

Cumulatively, it appears that modulation of cell wall invertase expression or activity represents a key step in regulating final nectar quality across species. If indeed sucrose transport across the plasma membrane is fully concentration‐dependent (passive transport via SWEET9), and the final nectar sucrose concentration is >2 M in squash (Table S2), then cytosolic sucrose in the nectary parenchyma must reach at least the concentration present in the nectar. This secretory process would present a tremendous osmotic stress to the nectary tissue, which may be why senescence‐related transcripts are upregulated in post‐secretory (+9 hr) nectaries (Appendix [Link], [Link], [Link]). A model of the approximate timing for the transition from starch synthesis to starch degradation to sucrose synthesis and export are shown in Figure 7c; since post‐secretory hydrolysis likely plays a limited role in the secretory process, CpCWINV4 activity is denoted with a light dashed line.

As previously noted, nectars are much more complex than simple sugar–water, containing many classes of biologically relevant solutes that influence not only pollinator visitation, but also impact microbial growth (Nicolson & Thornburg, 2007; Roy et al., 2017). In this study, we reliably identified over 40 metabolites in both male and female nectar, which is similar to an analysis of *C. maxima* (pumpkin) nectar (Chatt et al., 2018). While male nectar preferentially accumulates certain metabolites as compared to female nectar (Figure 6), the causes or consequences of these differences are not clear. However, there were some notable findings within the metabolite analysis, including the fact that female nectar contained ~5‐fold more GABA than male nectar, which was correlated to the expression of a putative GABA transporter (Figure S1). GABA is a neurotransmitter previously reported to accumulate in squash nectar (Nepi et al., 2012), but its role in plant–pollinator interactions is currently unclear. Along these lines, we identified another previously unreported neurotransmitter, gamma‐hydroxybutyric acid, in both male and female nectars, the role of which in nectar is also unknown. Of notable interest will be to determine factors that lead to differential transcription between female and male nectaries that may lead to differences in nectar quality. In our study *MYB305*, a key transcription factor in eudicot floral nectary function (Liu & Thornburg, 2012; Liu et al., 2009; Schmitt et al., 2018; Wang et al., 2014), displayed little difference in expression between squash male and female nectaries; however, three transcription factors*—SHI‐related sequence 1*,* heat stress transcription factor A‐6b,* and *UNUSUAL FLORAL ORGANS‐like—*displayed preferential expression female over male nectaries. Interestingly, *SHI‐related sequence 1* is a member of the *STYLISH* gene family, which control nectary development in Aquilegia (Min, Bunn, & Kramer, 2018).

To summarize, our analyses have revealed a distinct profile of starch biosynthetic and degradative pathways as the nectaries progress through maturation. Our expression data reveal a similar increase in sucrose synthesis in nectaries at anthesis, as well as a number of Arabidopsis gene homologs known to impact nectary function. We provide a physiological basis behind our gene expression data with starch, amylase, and soluble sugar assays showing degradation of starch correlated with increased amylase activity and accumulation of soluble sugar. Cumulatively, our data supports the existing model of nectar secretion in the eudicots (Lin et al., 2014; Roy et al., 2017). Lastly, we have identified novel nectar metabolites that should be evaluated for their roles in plant‐biotic interactions. This study represents an important step toward improving our understanding of nectar production and secretion in the cucurbits.

## CONFLICT OF INTEREST

The authors declare no conflict of interest associated with the work described in this manuscript.

## AUTHOR CONTRIBUTION

All authors conceived and planned the study. PMK determined nectary stages and conducted RNA isolations. MH processed all RNAseq data and conducted informatics analyses in conjunction with ES, RR, and CJC. ES and RR conducted starch and sugar analyses. ES conducted the qRT‐PCR analyses. EC and BJN conducted the metabolomic analyses. ES, RR, and CJC wrote the manuscript with feedback from all the coauthors.

## Data Availability

Raw sequence reads are available at the National Center for Biotechnology Information Sequence Read Archive under GEO accession number GSE111695.

## References

[pld3120-bib-0001] Adler, L. S. , & Irwin, R. E. (2012). Nectar alkaloids decrease pollination and female reproduction in a native plant. Oecologia, 168, 1033–1041. 10.1007/s00442-011-2153-3 22011842

[pld3120-bib-0002] Altschul, S. F. , Gish, W. , Miller, W. , Myers, E. W. , & Lipman, D. J. (1990). Basic local alignment search tool. Journal of Molecular Biology, 215, 403–410. 2231712 10.1016/S0022-2836(05)80360-2

[pld3120-bib-0003] Anders, S. , & Huber, W. (2010). Differential expression analysis for sequence count data. Genome Biology, 11, R106. 20979621 10.1186/gb-2010-11-10-r106 PMC3218662

[pld3120-bib-0004] Bender, R. L. , Fekete, M. L. , Klinkenberg, P. M. , Hampton, M. , Bauer, B. , Malecha, M. , … Carter, C. J. (2013). *PIN6* is required for nectary auxin response and short stamen development. The Plant Journal, 74, 893–904. 23551385 10.1111/tpj.12184

[pld3120-bib-0005] Bender, R. , Klinkenberg, P. , Jiang, Z. , Bauer, B. , Karypis, G. , Nguyen, N. , … Carter, C. J. (2012). Functional genomics of nectar production in the Brassicaceae. Flora ‐ Morphology, Distribution, Functional Ecology of Plants, 207, 491–496. 10.1016/j.flora.2012.06.005

[pld3120-bib-0006] Benjamini, Y. , & Hochberg, Y. (1995). Controlling the false discovery rate ‐ A practical and powerful approach to multiple testing. Journal of the Royal Statistical Society: Series B (Methodological), 57, 289–300. 10.1111/j.2517-6161.1995.tb02031.x

[pld3120-bib-0007] Boeker, P. , Leppert, J. , Mysliwietz, B. , & Lammers, P. S. (2013). Comprehensive theory of the Deans’ Switch as a variable flow splitter: Fluid mechanics, mass balance, and system behavior. Analytical Chemistry, 85, 9021–9030. 10.1021/ac401419j 23980824

[pld3120-bib-0008] Carmi, N. , Zhang, G. , Petreikov, M. , Gao, Z. , Eyal, Y. , Granot, D. , & Schaffer, A. A. (2003). Cloning and functional expression of alkaline alpha‐galactosidase from melon fruit: Similarity to plant SIP proteins uncovers a novel family of plant glycosyl hydrolases. The Plant Journal, 33, 97–106. 10.1046/j.1365-313X.2003.01609.x 12943544

[pld3120-bib-0009] Carter, C. , Graham, R. A. , & Thornburg, R. W. (1999). Nectarin I is a novel, soluble germin‐like protein expressed in the nectar of *Nicotiana* sp. Plant Molecular Biology, 41, 207–216. 10.1023/A:1006363508648 10579488

[pld3120-bib-0010] Carter, C. , Healy, R. , O'Tool, N. M. , Naqvi, S. M. , Ren, G. , Park, S. , … Thornburg, R. W. (2007). Tobacco nectaries express a novel NADPH oxidase implicated in the defense of floral reproductive tissues against microorganisms. Plant Physiology, 143, 389–399. 17114277 10.1104/pp.106.089326 PMC1761964

[pld3120-bib-0011] Carter, C. , Shafir, S. , Yehonatan, L. , Palmer, R. G. , & Thornburg, R. (2006). A novel role for proline in plant floral nectars. Naturwissenschaften, 93, 72–79. 10.1007/s00114-005-0062-1 16365739

[pld3120-bib-0012] Carter, C. , & Thornburg, R. W. (2000). Tobacco nectarin I. Purification and characterization as a germin‐like, manganese superoxide dismutase implicated in the defense of floral reproductive tissues. Journal of Biological Chemistry, 275, 36726–36733. 10.1074/jbc.M006461200 10952990

[pld3120-bib-0013] Carter, C. , & Thornburg, R. W. (2003). The nectary‐specific pattern of expression of the tobacco Nectarin I promoter is regulated by multiple promoter elements. Plant Molecular Biology, 51, 451–457. 10.1023/A:1022370203570 12650612

[pld3120-bib-0014] Carter, C. , & Thornburg, R. W. (2004a). Is the nectar redox cycle a floral defense against microbial attack? Trends in Plant Science, 9, 320–324. 10.1016/j.tplants.2004.05.008 15231276

[pld3120-bib-0015] Carter, C. J. , & Thornburg, R. W. (2004b). Tobacco Nectarin III is a bifunctional enzyme with monodehydroascorbate reductase and carbonic anhydrase activities. Plant Molecular Biology, 54, 415–425. 10.1023/B:PLAN.0000036373.84579.13 15284496

[pld3120-bib-0016] Carter, C. J. , & Thornburg, R. W. (2004c). Tobacco nectarin V is a flavin‐containing berberine bridge enzyme‐like protein with glucose oxidase activity. Plant Physiology, 134, 460–469. 10.1104/pp.103.027482 14730073 PMC316325

[pld3120-bib-0017] Chatt, E. C. , von Aderkas, P. , Carter, C. J. , Smith, D. , Elliott, M. , & Nikolau, B. J. (2018). Sex‐dependent vriation of pumpkin (*Cucurbita maxima* cv. Big Max) nectar and nectaries as determined by proteomics and metabolomics. Frontiers in Plant Science, 9, 860. 30008725 10.3389/fpls.2018.00860 PMC6034135

[pld3120-bib-0018] Davis, A. R. , Fowke, L. C. , Sawhney, V. K. , & Low, N. H. (1996). Floral nectar secretion and ploidy in *Brassica rapa* and *B. napus* (Brassicaceae) II. Quantified variability of nectary structure and function in rapid‐cycling lines. Annals of Botany, 77, 223–234. 10.1006/anbo.1996.0026

[pld3120-bib-0019] Davis, A. R. , Pylatuik, J. D. , Paradis, J. C. , & Low, N. H. (1998). Nectar‐carbohydrate production and composition vary in relation to nectary anatomy and location within individual flowers of several species of Brassicaceae. Planta, 205, 305–318. 10.1007/s004250050325 9637073

[pld3120-bib-0020] Davis, A. R. , Sawhney, V. K. , Fowke, L. C. , & Low, N. L. (1995). Floral secretion and ploidy in Brassica rapa and B napus (Brassicaceae).1. Nectary size and nectar carbohydrate production and composition (vol 25, pg 602, 1994). Apidologie, 26, 534.

[pld3120-bib-0021] Dmitruk, M. , & Weryszko‐Chmielewska, E. (2013). The morphology and ultrastructure of the nectaries of marrow (*Cucurbita pepo* L. convar. giromontiina). Acta Agrobotanica, 66, 11–22. 10.5586/aa.2013.033

[pld3120-bib-0022] Elliott, S. E. , Irwin, R. E. , Adler, L. S. , & Williams, N. M. (2008). The nectar alkaloid, gelsemine, does not affect offspring performance of a native solitary bee, Osmia lignaria (Megachilidae). Ecological Entomology, 33, 298–304. 10.1111/j.1365-2311.2007.00974.x

[pld3120-bib-0023] Grabherr, M. G. , Haas, B. J. , Yassour, M. , Levin, J. Z. , Thompson, D. A. , Amit, I. , … Regev, A. (2011). Full‐length transcriptome assembly from RNA‐Seq data without a reference genome. Nature Biotechnology, 29, 644–652. 10.1038/nbt.1883 PMC3571712 21572440

[pld3120-bib-0024] Hampton, M. , Xu, W. W. , Kram, B. W. , Chambers, E. M. , Ehrnriter, J. S. , Gralewski, J. H. , … Carter, C. J. (2010). Identification of differential gene expression in *Brassica rapa* nectaries through expressed sequence tag analysis. PLoS ONE, 5, e8782 10.1371/journal.pone.0008782 20098697 PMC2808342

[pld3120-bib-0025] Heil, M. (2011). Nectar: Generation, regulation and ecological functions. Trends in Plant Science, 16, 191–200. 10.1016/j.tplants.2011.01.003 21345715

[pld3120-bib-0026] Heil, M. , Koch, T. , Hilpert, A. , Fiala, B. , Boland, W. , & Linsenmair, K. (2001). Extrafloral nectar production of the ant‐associated plant, Macaranga tanarius, is an induced, indirect, defensive response elicited by jasmonic acid. Proceedings of the National Academy of Sciences of the United States of America, 98, 1083–1088. 10.1073/pnas.98.3.1083 11158598 PMC14712

[pld3120-bib-0027] Horner, H. T. , Healy, R. A. , Ren, G. , Fritz, D. , Klyne, A. , Seames, C. , & Thornburg, R. W. (2007). Amyloplast to chromoplast conversion in developing ornamental tobacco floral nectaries provides sugar for nectar and antioxidants for protection. American Journal of Botany, 94, 12–24. 10.3732/ajb.94.1.12 21642203

[pld3120-bib-0028] Irwin, R. E. , & Adler, L. S. (2008). Nectar secondary compounds affect self‐pollen transfer: Implications for female and male reproduction. Ecology, 89, 2207–2217. 10.1890/07-1359.1 18724731

[pld3120-bib-0029] Irwin, R. E. , Adler, L. S. , & Agrawal, A. A. (2004). Community and evolutionary ecology of nectar. Ecology, 85, 1477–1478. 10.1890/03-0717

[pld3120-bib-0030] Kram, B. W. , & Carter, C. J. (2009). *Arabidopsis thaliana* as a model for functional nectary analysis. Sexual Plant Reproduction, 22, 235–246. 10.1007/s00497-009-0112-5 20033445

[pld3120-bib-0031] Kram, B. W. , Xu, W. W. , & Carter, C. J. (2009). Uncovering the *Arabidopsis thaliana* nectary transcriptome: Investigation of differential gene expression in floral nectariferous tissues. BMC Plant Biology, 9, 92 10.1186/1471-2229-9-92 19604393 PMC2720969

[pld3120-bib-0032] Lin, I. W. , Sosso, D. , Chen, L. Q. , Gase, K. , Kim, S. G. , Kessler, D. , … Frommer, W. B. (2014). Nectar secretion requires sucrose phosphate synthases and the sugar transporter SWEET9. Nature, 508, 546–549. 10.1038/nature13082 24670640

[pld3120-bib-0033] Liu, G. , Ren, G. , Guirgis, A. , & Thornburg, R. W. (2009). The MYB305 transcription factor regulates expression of nectarin genes in the ornamental tobacco floral nectary. The Plant Cell, 21, 2672–2687. 10.1105/tpc.108.060079 19783761 PMC2768911

[pld3120-bib-0034] Liu, G. , & Thornburg, R. W. (2012). Knockdown of MYB305 disrupts nectary starch metabolism and floral nectar production. The Plant Journal, 70, 377–388. 10.1111/j.1365-313X.2011.04875.x 22151247

[pld3120-bib-0035] Mi, H. , Huang, X. , Muruganujan, A. , Tang, H. , Mills, C. , Kang, D. , & Thomas, P. D. (2017). PANTHER version 11: Expanded annotation data from Gene Ontology and Reactome pathways, and data analysis tool enhancements. Nucleic Acids Research, 45, D183–D189. 10.1093/nar/gkw1138 27899595 PMC5210595

[pld3120-bib-0036] Min, Y. , Bunn, J. I. , & Kramer, E. M. (2018). Homologs of the STYLISH gene family control nectary development in Aquilegia. New Phytologist, 221, 1090–1100. 30145791 10.1111/nph.15406

[pld3120-bib-0037] Montero‐Pau, J. , Blanca, J. , Bombarely, A. , Ziarsolo, P. , Esteras, C. , Marti‐Gomez, C. , … Canizares, J. (2018). De novo assembly of the zucchini genome reveals a whole‐genome duplication associated with the origin of the Cucurbita genus. Plant Biotechnology Journal, 16, 1161–1171. 10.1101/147702 29112324 PMC5978595

[pld3120-bib-0038] Naqvi, S. M. , Harper, A. , Carter, C. , Ren, G. , Guirgis, A. , York, W. S. , & Thornburg, R. W. (2005). Nectarin IV, a potent endoglucanase inhibitor secreted into the nectar of ornamental tobacco plants. Isolation, cloning, and characterization. Plant Physiology, 139, 1389–1400. 10.1104/pp.105.065227 16244157 PMC1283774

[pld3120-bib-0039] Nepi, M. , Guarnieri, M. , & Pacini, E. (2001). Nectar secretion, reabsorption, and sugar composition in male and female flowers of *Cucurbita pepo* . International Journal of Plant Sciences, 162, 353–358. 10.1086/319581

[pld3120-bib-0040] Nepi, M. , Pacini, E. , & Willemse, M. T. M. (1996). Nectary biology of *Cucurbita pepo*: Ecophysiological aspects. Acta Botanica Neerlandica, 45, 41–54. 10.1111/j.1438-8677.1996.tb00494.x

[pld3120-bib-0041] Nepi, M. , Soligo, C. , Nocentini, D. , Abate, M. , Guarnieri, M. , Cai, G. , … Pacini, E. (2012). Amino acids and protein profile in floral nectar: Much more than a simple reward. Flora ‐ Morphology, Distribution, Functional Ecology of Plants, 207, 475–481. 10.1016/j.flora.2012.06.002

[pld3120-bib-0042] Nepi, M. , & Stpiczynska, M. (2008). The complexity of nectar: Secretion and resorption dynamically regulate nectar features. Naturwissenschaften, 95, 177–184. 10.1007/s00114-007-0307-2 17922103

[pld3120-bib-0043] Nicolson, S. , & Thornburg, R. (2007). Nectar chemistry In Nicolson S. W., Nepi M., & Pacini E. (Eds.), Nectaries and nectar (p. 395). Dordrecht, The Netherlands: Springer.

[pld3120-bib-0044] Ollerton, J. , Winfree, R. , & Tarrant, S. (2011). How many flowering plants are pollinated by animals? Oikos, 120, 321–326. 10.1111/j.1600-0706.2010.18644.x

[pld3120-bib-0045] Pacini, E. , & Nepi, M. (2007). Nectar production and presentation In Nicolson S. W., Nepi M., & Pacini E. (Eds.), Nectaries and nectar (pp. 167–214). Dordrecht, The Netherlands: Springer 10.1007/978-1-4020-5937-7

[pld3120-bib-0046] Peng, Y. B. , Li, Y. Q. , Hao, Y. J. , Xu, Z. H. , & Bai, S. N. (2004). Nectar production and transportation in the nectaries of the female Cucumis sativus L. flower during anthesis. Protoplasma, 224, 71–78. 15726811 10.1007/s00709-004-0051-9

[pld3120-bib-0047] Pleasants, J. M. , & Chaplin, S. J. (1983). Nectar production rates of Asclepias quadrifolia: Causes and consequences of individual variation. Oecologia, 59, 232–238. 10.1007/BF00378842 28310238

[pld3120-bib-0048] Prasifka, J. R. , Mallinger, R. E. , Portlas, Z. M. , Hulke, B. S. , Fugate, K. K. , Paradis, T. , … Carter, C. J. (2018). Using nectar‐related traits to enhance crop‐pollinator interactions. Frontiers in Plant Science, 9, 812 10.3389/fpls.2018.00812 29967631 PMC6015894

[pld3120-bib-0049] Radhika, V. , Kost, C. , Boland, W. , & Heil, M. (2010). The role of jasmonates in floral nectar secretion. PLoS ONE, 5, e9265 10.1371/journal.pone.0009265 20174464 PMC2824824

[pld3120-bib-0050] Ren, G. , Healy, R. A. , Horner, H. T. , Martha, G. J. , & Thornburg, R. W. (2007). Expression of starch metabolic genes in the developing nectaries of ornamental tobacco plants. Plant Science, 173, 621–637. 10.1016/j.plantsci.2007.08.012

[pld3120-bib-0051] Ren, G. , Healy, R. A. , Klyne, A. M. , Horner, H. T. , James, M. G. , & Thornburg, R. W. (2007). Transient starch metabolism in ornamental tobacco floral nectaries regulates nectar composition and release. Plant Science, 173, 277–290. 10.1016/j.plantsci.2007.05.008

[pld3120-bib-0052] Richardson, L. L. , Adler, L. S. , Leonard, A. S. , Andicoechea, J. , Regan, K. H. , Anthony, W. E. , … Irwin, R. E. (2015). Secondary metabolites in floral nectar reduce parasite infections in bumblebees. Proceedings of the Royal Society B: Biological Sciences, 282, 20142471. 10.1098/rspb.2014.2471 PMC4345440 25694627

[pld3120-bib-0053] Roy, R. , Schmitt, A. J. , Thomas, J. B. , & Carter, C. J. (2017). Review: Nectar biology: From molecules to ecosystems. Plant Science, 262, 148–164. 10.1016/j.plantsci.2017.04.012 28716410

[pld3120-bib-0054] Ruhlmann, J. M. , Kram, B. W. , & Carter, C. J. (2010). *CELL WALL INVERTASE 4* is required for nectar production in Arabidopsis. Journal of Experimental Botany, 61, 395–404. 10.1093/jxb/erp309 19861655 PMC2803206

[pld3120-bib-0055] Schapendonk, A. H. C. M. , & Brouwer, P. (1984). Fruit‐growth of cucumber in relation to assimilate supply and sink activity. Scientia Horticulturae, 23, 21–33. 10.1016/0304-4238(84)90041-4

[pld3120-bib-0056] Schmitt, A. J. , Roy, R. , Klinkenberg, P. M. , Jia, M. Y. , & Carter, C. J. (2018). The octadecanoid pathway, but not COI1, is required for nectar secretion in *Arabidopsis thaliana* . Frontiers in Plant Science, 9, 1060. 30135692 10.3389/fpls.2018.01060 PMC6092685

[pld3120-bib-0057] Simpson, B. B. , & Neff, J. L. (1983). Evolution and diversity of floral rewards In Jones C. E., & Little R. J. (Eds.), Handbook of experimental pollination biology (pp. 142–159). New York, NY: Van Nostrand Reinhold.

[pld3120-bib-0058] Stitz, M. , Hartl, M. , Baldwin, I. T. , & Gaquerel, E. (2014). Jasmonoyl‐L‐Isoleucine coordinates metabolic networks required for anthesis and floral attractant emission in wild tobacco (*Nicotiana attenuata*). The Plant Cell, 26, 3964–3983. 10.1105/tpc.114.128165 25326292 PMC4247565

[pld3120-bib-0059] Streb, S. , & Zeeman, S. C. (2012). Starch metabolism in Arabidopsis. The Arabidopsis Book, 10, e0160 10.1199/tab.0160 23393426 PMC3527087

[pld3120-bib-0060] Thomas, J. B. , Hampton, M. , Dorn, K. M. , Marks, M. D. , & Carter, C. J. (2017). The pennycress (*Thlaspi arvense* L. Brassicaceae) nectary: Structural and transcriptomic characterization. BMC Plant Biology, 17, 201. 29137608 10.1186/s12870-017-1146-8 PMC5686818

[pld3120-bib-0061] Thornburg, R. W. , Carter, C. , Powell, A. , Mittler, R. , Rizhsky, L. , & Horner, H. T. (2003). A major function of the tobacco floral nectary is defense against microbial attack. Plant Systematics and Evolution, 238, 211–218. 10.1007/s00606-003-0282-9

[pld3120-bib-0062] Wang, W. , Liu, G. , Niu, H. , Timko, M. P. , & Zhang, H. (2014). The F‐box protein COI1 functions upstream of MYB305 to regulate primary carbohydrate metabolism in tobacco (Nicotiana tabacum L. cv. TN90). Journal of Experimental Botany, 65, 2147–2160. 10.1093/jxb/eru084 24604735 PMC3991746

[pld3120-bib-0063] Wiesen, L. B. , Bender, R. L. , Paradis, T. , Larson, A. , Perera, M. A. D. N. , Nikolau, B. J. , … Carter, C. J. (2016). A role for GIBBERELLIN 2‐OXIDASE6 and gibberellins in regulating nectar production. Molecular Plant, 9, 753–756. 10.1016/j.molp.2015.12.019 26748313

[pld3120-bib-0064] Zhang, B. , Tolstikov, V. , Turnbull, C. , Hicks, L. M. , & Fiehn, O. (2010). Divergent metabolome and proteome suggest functional independence of dual phloem transport systems in cucurbits. Proceedings of the National Academy of Sciences of the United States of America, 107, 13532–13537. 10.1073/pnas.0910558107 20566864 PMC2922161

[pld3120-bib-0065] Zhu, J. , Hu, Z. H. , & Müller, M. (1997). Ultrastructure of the floral nectary of Arabidopsis thaliana L. prepared from high pressure freezing and freeze substitution. Acta Botanica Sinica, 39, 289–295.

