# Supplementary figures and images for: An integrated transcriptomics and metabolomics analysis of the Cucurbita pepo nectary implicates key modules of primary metabolism involved in nectar synthesis and secretion
Source: Plant Direct. 2019 Feb 28;3(2):e00120. doi: 10.1002/pld3.120 (PMC6508809; doi:10.1002/pld3.120)

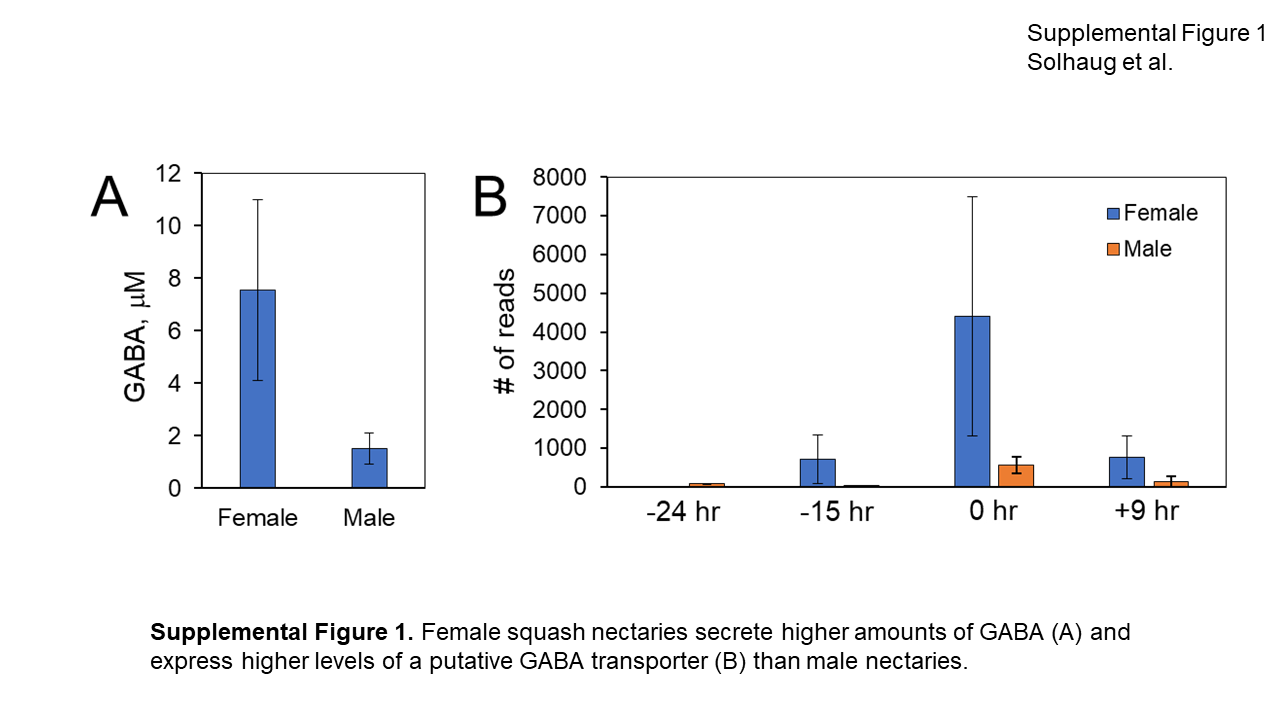

Supplement: Supplementary file 1 [file PLD3-3-e00120-s001.tif]
