# Supplementary material for: An integrated transcriptomics and metabolomics analysis of the Cucurbita pepo nectary implicates key modules of primary metabolism involved in nectar synthesis and secretion
Source: Plant Direct. 2019 Feb 28;3(2):e00120. doi: 10.1002/pld3.120 (PMC6508809; doi:10.1002/pld3.120)
Supplement: Supplementary file 15 [file PLD3-3-e00120-s015.pdf]

27 January 2019

Dear Editor and Reviewers:

We thank you for the thorough reading of the manuscript and agree with all of the comments for improvement. As such, we have addressed each of the concerns in the revised manuscript and associated supplemental files. Our responses are provided in blue text below.

Sincerely,

Clay Carter, corresponding author

==

1. Line 283. "Squash nectaries are considerably larger than Arabidopsis (~1 cm diameter for squash vs. ~100 µM for Arabidopsis)"

This statement does not make sense.

We have corrected this typo. Thank you for catching this.

This reviewer really thinks is the bioinformatics should be performed on zucchini genome (published in 2017). It is strange that the author did not discuss anything of zucchini genome in the manuscript. At least, the author should justify why they did not use the zucchini genome in bioinformatics and explain how the data can or cannot be improved.

We have now referenced the zucchini genome on line 317 and, per Reviewer #2's suggestion, incorporated the *C. pepo* orthologs of *C. melo* genes into the analysis. These IDs are now included in Supplemental Files 1-6 and 10-11. We apologize for the initial oversight.

2. Line 441. "Raffinose-family oligosaccharides (RFOs) are the primary transport sugars in cucurbits, which are hydrolyzed in sink tissues by a-galactosidases, releasing galactose and sucrose for further metabolism." Insert reference for this statement.

As requested, a citation has been added to the corresponding text (now on line 452). We also have taken this opportunity to add clarifying text.

3. Figure 4, PGM is classified as sucrose biosynthesis group, but considering expression pattern, it looks like it is plastid isoform of PGM important for starch biosynthesis?

We have added the following clarifying text beginning on line 433: "…with one notable exception being *phosphoglucomutase* (*CpPGM*), which was high at -24h, similar to other starch synthesis genes. PGM is involved in producing glucose-1P for both starch (via ADP-glucose) and sucrose synthesis (via UDP-glucose), and is likely important for both of these processes in *C. pepo* nectaries (Streb and Zeeman, 2012)."

This text was added to clarify that the genes were grouped based on their involvement in certain processes, not because they exhibited similar expression patterns. The support for our hypotheses of induction of these processes comes from comparing the expression patterns of these genes, which were selected based on the Streb and Zeeman review (2012) that is now cited.

==

Reviewer #2:

My only concern with the manuscript is that it ignores the C. pepo genome (as also noted by reviewer 1) published by Montero-Pau et al (2018) [Plant Biotechnol J, 16: 1161-117]. I imagine that it is likely that the initial transcriptome analysis including contig assembly and annotation may have been done previous to the release of the annotated genome, but since the release happened in 2017 (https://onlinelibrary.wiley.com/doi/full/10.1111/pbi.12860) it will be appropriate to at least include a table of equivalences between the differentially expressed contigs annotated in this manuscript and the genes annotated by Montero-Pau et al, and include proper references to that work. This could be included as another field in supplemental files 3-6 (likely more useful), or as another supplemental file combining all DE genes.

Please see our response to Reviewer #1's first comment above. Thank you.
